# Research hotspots and trends in the relationship between sport and nutrition: A bibliometric analysis from 2013 to 2023

**DOI:** 10.1097/MD.0000000000037782

**Published:** 2024-04-19

**Authors:** Ye Tao, Wenqiang Wu

**Affiliations:** a Department of Physical Education, Beijing University of Posts and Telecommunications, Beijing, P.R. China; b Dean of China Athletics College, Beijing Sport University, Beijing, P.R. China.

**Keywords:** bibliometric analysis, Citespace, knowledge mapping, nutrition, sport

## Abstract

This research aimed to summarize the research development and hot points in on the connection between sport and nutrition overall through bibliometric analysis. We collected the publications in the last 10 years (2013–2023) related to between sport and nutrition in the Web of Science database, and applied Citespace to assess the knowledge mapping. The results showed as follows that the number of manuscripts about sport and nutrition totaled 10,016, with a faster increase after 2019. The country, institution, and author with the most publications are the USA, University of California System, Burke, Louise M. In addition, the most co-cited reference is *Journal of the Academy of Nutrition and Dietetics* (2016) (199). Based on a 10-year bibliometric investigation, we know the USA, the University of California System has become one of this discipline’s major research forces. Research on sport and nutrition benefits from the best partnerships between industrialized nations and prominent universities.

## 1. Introduction

The adaptive response to exercise training is determined by a combination of factors: the duration, the intensity, and the type of exercise as well as the frequency of training, but also by the quality and quantity of nutrition in the pre- and postexercise periods. The foundation of an athlete’s diet is an adequate energy intake since it promotes optimal bodily function, establishes the athlete’s capacity to consume macro- and micronutrients, and helps to manipulate body composition. It is becoming more and more obvious that nutrition can either enhance or reduce adaptations that are triggered by exercise. For instance, it is commonly known that when there is no protein feeding following exercise, there is little net protein synthesis and the muscle may even be in negative protein balance. Additionally, there is proof that reducing the availability of carbohydrates can encourage particular muscle adaptations. On the other hand, high-concentration antioxidant supplements may lessen the effects of training adaptations.^[1–3]^ The majority of research has been on skeletal muscle adaptations. Crucially, a variety of additional organ adaptations that are crucial to athletic performance and are impacted by dietary intake exist. These modifications and their significance for athletes are frequently disregarded or have gotten comparatively little attention. The intestine, the brain, and the vasculature are a few examples, but there are many. For instance, there is proof that the gut’s glucose transporters are upregulated in reaction to carbohydrate eating, and that dietary changes can modify the gut’s microflora. Such adjustments might modify how nutrients are delivered and so impact performance. Long-term exercise performance outcomes are thus ultimately determined by a multitude of interactions between exercise and nutrition as well as a multitude of impacts of nutrition alone.

Understanding the latest developments in sports and nutrition science is crucial. The field of sport and nutrition research is developing so quickly that it is challenging to properly understand its current state and hotspots. The quantitative study of particular fields utilizing a range of databases, such as Pubmed, Web of Science (WOS), etc, is known as bibliometric.^[4,5]^ Citespace program allows WOS to perform scientific quantitative analysis among them.^[6,7]^ Readers can completely understand the frontiers, trends, and hotspots in this industry with the use of bibliometrics.^[4,6]^ It examines the pivotal moments in the development of subject areas. coauthor, co-citation, and co-occurrence analysis are examples of bibliometrics.^[8,9]^

There are still a lot of gaps in the literature, despite tremendous progress in our understanding of the dietary needs of athletes. Nutrition research is still a complicated field that is always changing and even contradicting. Sports medicine, sports science, dietetics, cultural influences, and even popular media are all included in the topic of “sports nutrition.” Healthcare professionals such as nutritionists, registered dietitians, sports scientists, physicians, and others frequently disagree on the optimal diet, particularly when it comes to athletes and specifically “what to eat.”^[10,11]^ Given these circumstances, this illness has received increased attention from the nutrition and sports domains, resulting in the widespread publication of research results as scholarly articles. On the one hand, the increase in published research has greatly accelerated progress in the field of sport and nutrition collaborations. However, it is still difficult to get a thorough grasp of the status of research on nutrition and sport today because of the large number of scattered, sometimes duplicate articles that make up this field. Currently, there is no bibliometric approach for studying the relationship between nutrition and sport. In order to achieve this, Citespace was utilized to examine the global significance and patterns of nutrition and sport in the WOS database between January 1, 2013, and November 30, 2023.

## 2. Method

### 2.1. Source of literature

We enter the subject terms into the WOS database (SCI, SSCI, ESCI, etc): TS = (exercise OR sport OR sports OR sport player) AND (nutrition OR nourishment). The search scope of the database is from January 1, 2013 to November 30, 2023, and the language type was English. Through the literature search (articles, reviews, meeting abstracts, etc), 14,883 records were obtained. The WOS database comes from the Beijing University of Posts and Telecommunications database in China. All the articles containing “search terms” have been checked (because some retrieved articles may not necessarily be 100% relevant). Two researchers worked independently to screen the literature and assess its quality. These publications provided bibliometric information, such as the publication date, the yearly quantity of publications/citations, the countries/regions, the institutions, the authors, the journals, the references, and the keywords.

### 2.2. Analysis software

Citespace analysis software Version 5.6. R2.^[12]^

### 2.3. Download and import of data

The results of the retrieved subject terms were exported, and the file format was kept as “plain text.”

### 2.4. Parameter setting

Time slicing (from 2013 to 2023); node type (checked individually); 50 selection criteria; trimming (pathfinder); and visualization (showing the merged network, cluster view-static).

### 2.5. Statistical methods

**Figure 1.**
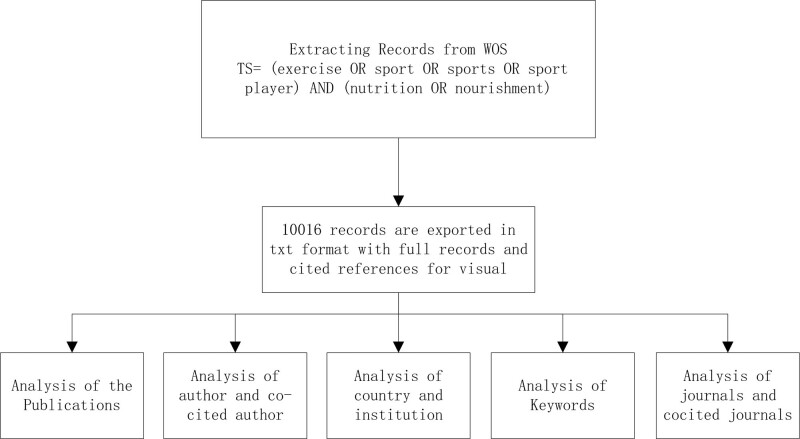
Analysis flow chart of sport and nutrition.

Microsoft Office Excel 2021 was used to manage data and analyze annual publications. All literature has been scientifically analyzed; Some data obtained include core countries, institutions, authors, co-cited references, keyword co-occurrence.^[13–15]^ The detailed analysis flow is shown in Figure 1.

## 3. Results

### 3.1. Analysis of the publications

**Figure 2.**
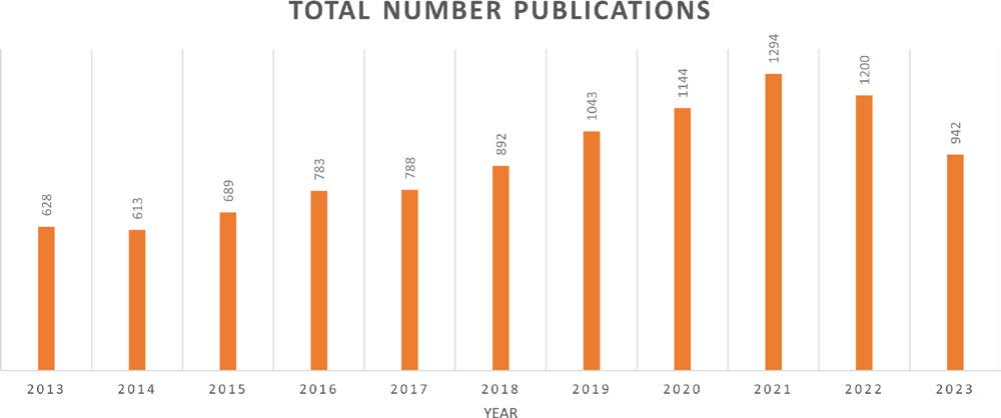
The number of sport and nutrition publications indexed by WOS from 2013 to 2023.

Over the course of the research period, the total number of papers rose and fell. As illustrated in Figure 2, the research is divided into 2 stages: the first stage covers 2013 to 2018, and the second stage covers 2019 to 2023. The second stage was a period of rapid development. The publication published 892 references in 2018, rising to 1043 references in 2019. In 2021, the number increased to 1294. These findings suggest that throughout the previous 5 years, research on sport and nutrition has grown in importance.

### 3.2. Analysis of countries and institutions

**Figure 3.**
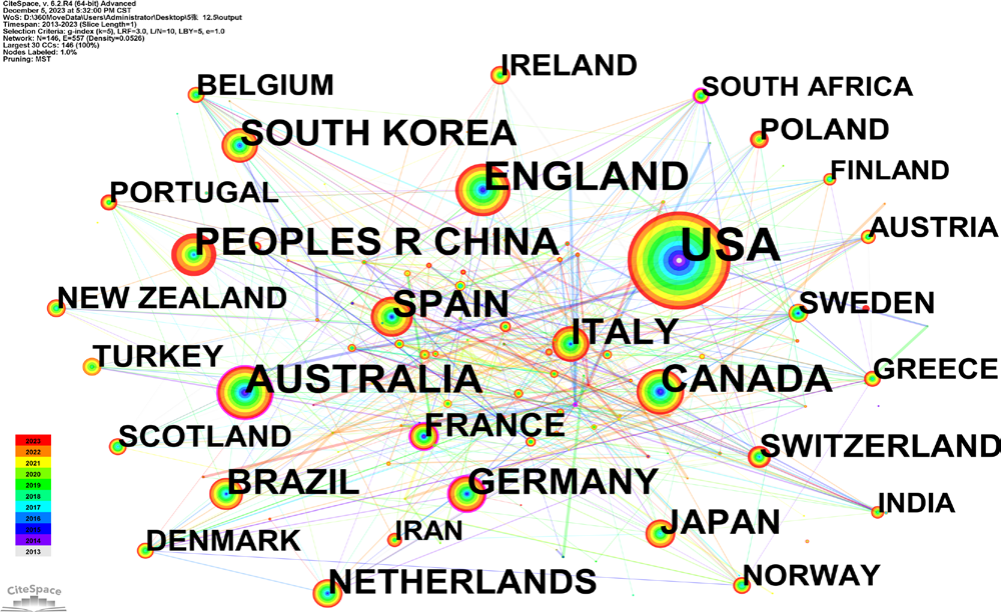
Analysis of the country map from 2013 to 2023.

A country map was generated (Fig. 3). One hundred forty-seven countries published 10,011 references. The USA, England, Australia, Canada, and People’s R China are the top 5 countries (Table 1). The Australia (0.11) and Germany (0.11) are the top 2 countries from centrality (purple round). An analysis of publications and centrality shows that the USA, People’s R China, and France were the main research forces in the study of the relationship between sport and nutrition. Spain, Italy, and South Korea have been increasingly interested in this field. The majority of the research was conducted in industrialized nations, and there was little international cooperation.

**Table 1:** Top 5 countries and institutions researching sport and nutrition.

Ranking	Country	Publications	Ranking	Institution	Publications
1	USA	3306	1	University of California System	254
2	England	1042	2	Harvard University	234
3	Australia	957	3	University of London	203
4	Canada	782	4	University of Texas System	165
5	People’s R. China	724	5	University of North Carolina	147

**Figure 4.**
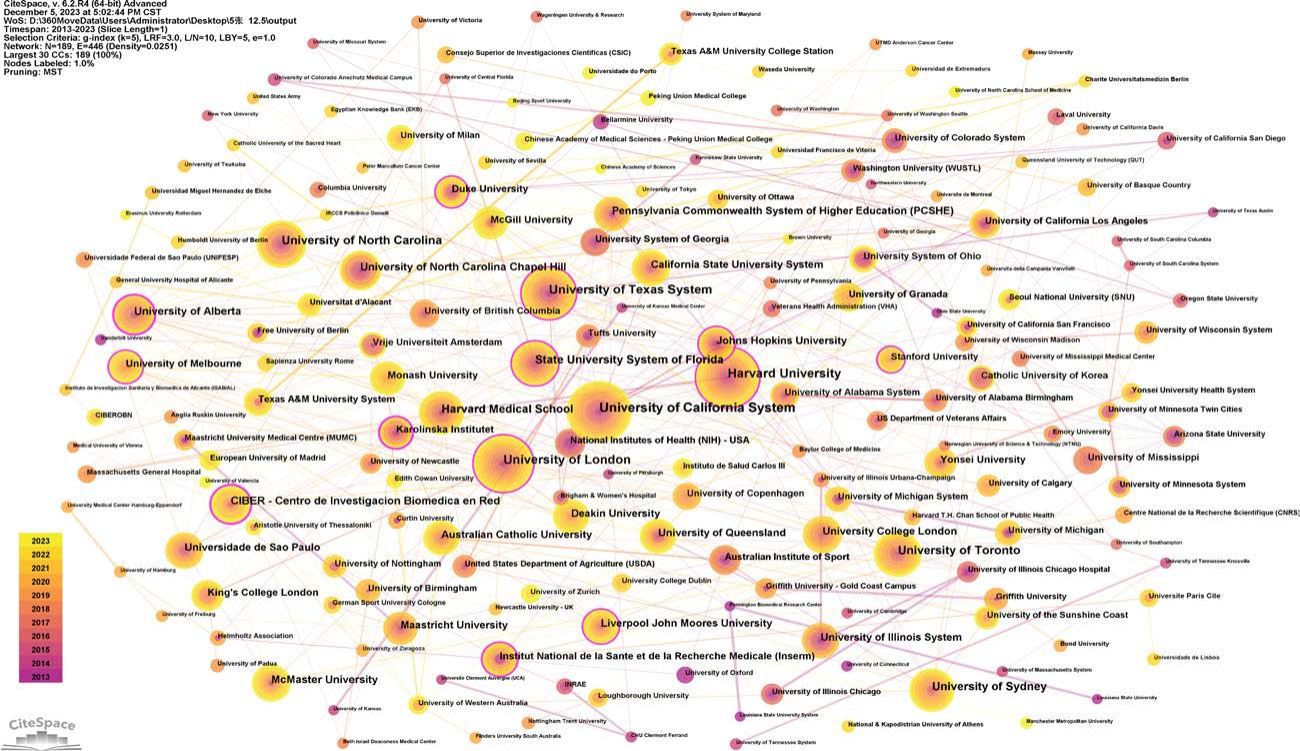
Institutional map researching sport and nutrition from 2013 to 2023.

Generated an institution map with 189 nodes and 446 links (Fig. 4). The 6152 publications have been published in 189 institutions. The University of California System, the Harvard University, University of London, University of Texas System, and the University of North Carolina) are the top 5 institutions (Table 1). Regarding centrality, the top 3 institutions were Karolinska Institutet (0.29), University of Alberta (0.28), and CIBER – Centro de Investigacion Biomedica en Red (0.21). Furthermore, the comparatively limited connections among different institutions point to a lack of cooperation.

### 3.3. Analysis of author

**Figure 5.**
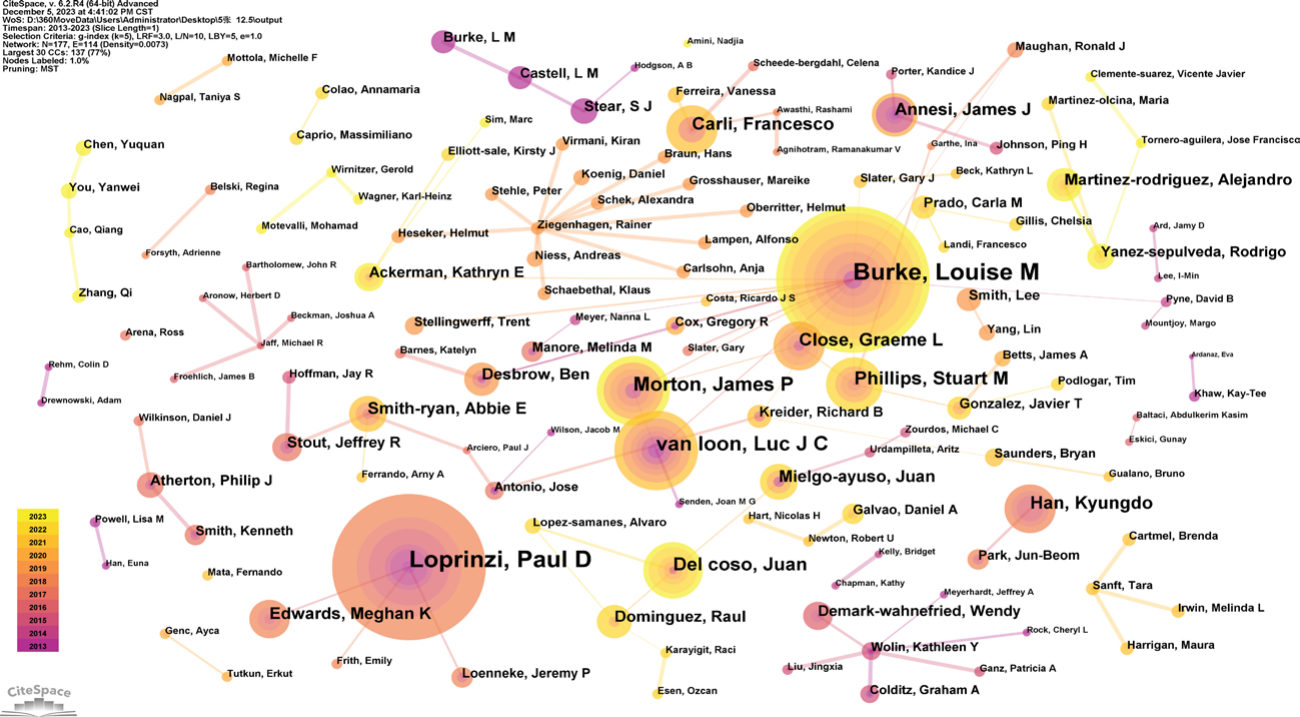
Author map researching sport and nutrition from 2013 to 2023.

One hundred seventy-seven authors published 1129 articles. The top 5 authors who have been written about (Table 2) are experts in this field. The generated map had 177 nodes and 114 links (Fig. 5).

**Table 2:** Top 5 authors in sports medicine of athletes research in terms of centrality.

Ranking	Cited reference	Centrality	Representative author (publication year)
1	57	0.08	Burke LM (2014)
2	57	0	Loprinzi PD (2013)
3	31	0	van Loon LJC (2013)
4	27	0	Morton JP (2015)
5	22	0	Del Coso J (2016)

As the writer whose works have been published the most, Louise M. Burke, is based at the Intra Performance Group. Their team could provide comprehensive support to clients, including performance nutrition, personal chefs, psychology and strategic consultancy^[12]^ (Table 2).

### 3.4. Analysis of co-cited references

**Figure 6.**
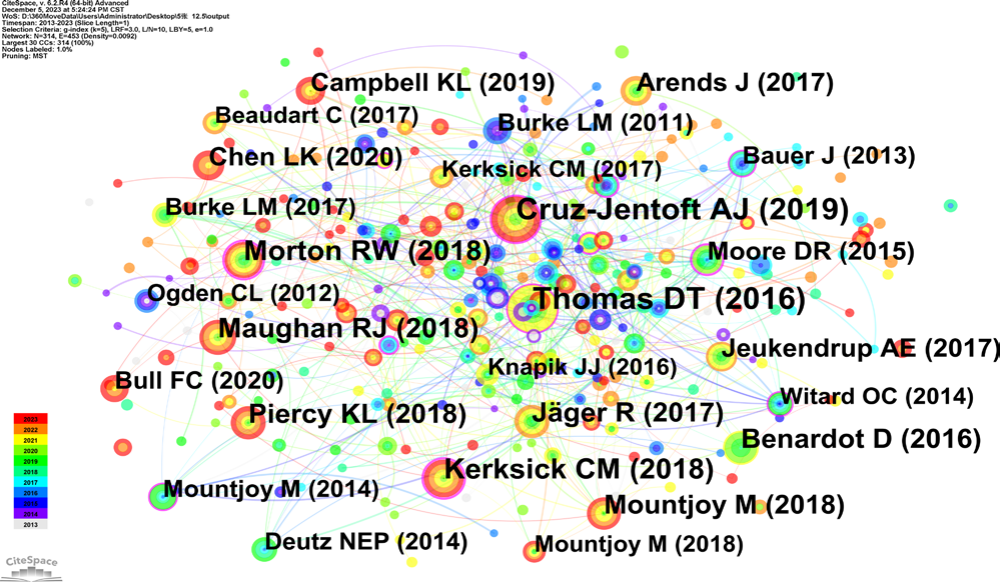
Co-cited references map researching sport and nutrition from 2013 to 2023.

An analysis of counts and centrality (Fig. 6, Table 3) revealed that the data usually comes in the form of a review. Among them, “Position of the Academy of Nutrition and Dietetics, Dietitians of Canada, and the American College of Sports Medicine: Nutrition and Athletic Performance” was published in *Journal of the Academy of Nutrition and Dietetics* in 2016. D. Travis Thomas experimentally confirmed that sporting activities are enhanced by well-chosen nutrition strategies. Furthermore, the distinct environmental elements of an individual have etiological relevance. Dietitians of Canada, the American College of Sports Medicine, and the Academy of Nutrition and Dietetics are the organizations for whom this position paper was created. It describes the positions taken by the Academy of Nutrition and Dietetics, Dietitians of Canada, and American College of Sports Medicine on dietary elements that have been shown to affect athletic performance as well as new developments in the field of sports nutrition. For a customized nutrition plan, athletes ought to be referred to a qualified dietitian nutritionist.^[13]^

**Table 3:** Top 5 co-cited references related to Sports medicine of athletes research in terms of co-citation.

Ranking	Cited reference	Co-citation counts	Representative author (publication year)
1	Position of the Academy of Nutrition and Dietetics, Dietitians of Canada, and the American College of Sports Medicine: Nutrition and Athletic Performance	199	Thomas DT (2016)
2	Sarcopenia: revised European consensus on definition and diagnosis	190	Cruz-Jentoft AJ (2019)
3	ISSN exercise & sports nutrition review update: research & recommendations	133	Kerksick CM (2018)
4	A systematic review, meta-analysis and meta-regression of the effect of protein supplementation on resistance training-induced gains in muscle mass and strength in healthy adults	117	Morton RW (2018)
5	IOC consensus statement: dietary supplements and the high-performance athlete	113	Maughan RJ (2018)

### 3.5. Analysis of keyword co-occurrence and burst analysis

**Figure 7.**
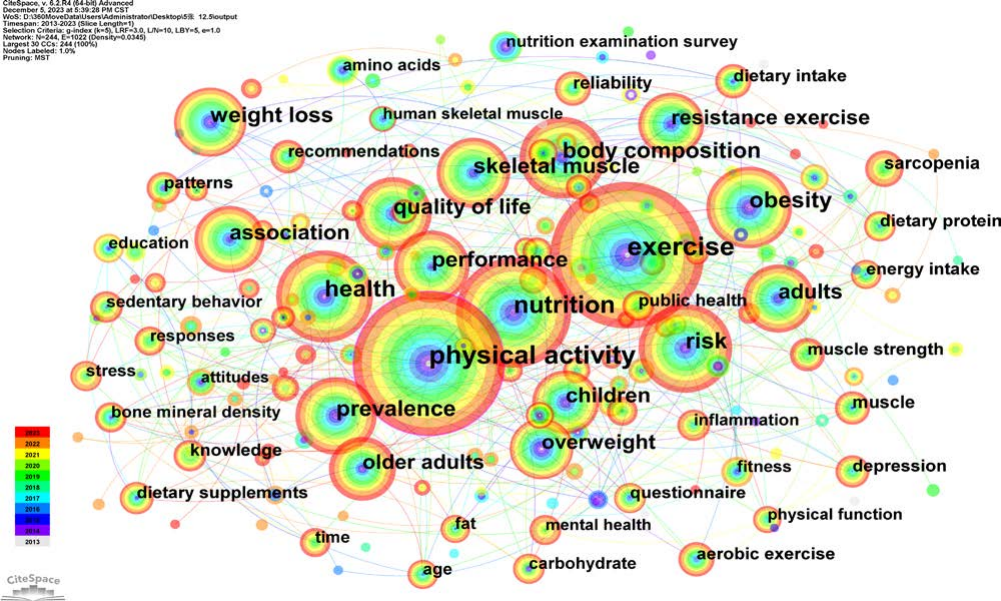
Co-occurrence keyword map researching sport and nutrition from 2013 to 2023.

A keyword co-occurrence map could reflect hot topics. Generated a keyword co-occurrence map had 244 nodes and 1022 links (Fig. 7). It shows that the prevalent keywords were exercise, physical activity, nutrition, health, and risk.

**Figure 8.**
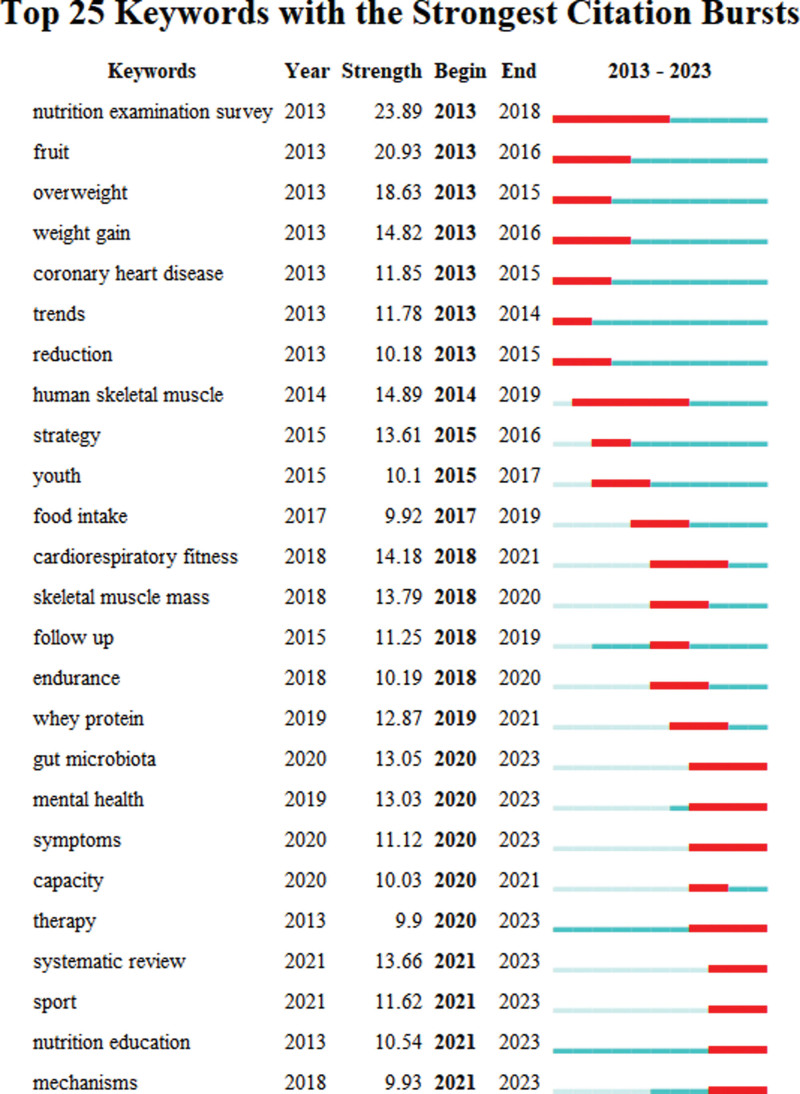
Top 25 keywords with the strongest citation bursts.

“Burst words” are those that are used a lot over an extended length of time. The distribution of terms with the strongest citation explosion could be used to anticipate the frontier of research trend. Figure 8 displays the top 25 terms with the strongest citation surge from 2013 to 2023. The green bars indicated infrequent citations of the keyword, whereas the red bars indicated frequent citations. Nutrition examination survey, fruit, overweight, weight gain, coronary heart disease, trends, reduction, human skeletal muscle, strategy, food intake, cardiorespiratory fitness may be quoted frequently, indicating the growing interest in this area of research in the upcoming years.

## 4. Discussion

According to Citespace’s data, there were more yearly articles regarding nutrition and sports between 2019 and 2023. It might be as a result of the growing emphasis that researchers are placing on nutrition, which is a crucial aspect of sports. Probably because of the publication of “ISSN exercise & sports nutrition review update: research & recommendations,”^[16]^ it has been cited 242 times. This article helps supporters and members of the International Society of Sports Nutrition stay informed on the most recent research in the field of sports nutrition. We found that the United States has the larger number of articles on sports nutrition, which may be due to the disadvantage of Chinese sports research in writing English articles.

The amount of nutrition studies on sport is growing, and scientists are paying more attention to the nutrition in sport. Individualized nutrition regimens for athletes must consider the particulars of the event, their performance objectives, real-world obstacles, food preferences, and how they react to different tactics. Dietetics is currently developing new paths that center on designing customized diets. Among these are: genetic tests that may ascertain an individual’s inclination toward a specific diet and the level of risk associated with food-related illnesses^[14]^; studies on the variety of the human microbiota, the features of digestion, and the condition of the intestinal barrier^[15,17]^; and investigations of individual immune system responses to food antigens that alter food tolerance and the reactivity of the adaptive immune response. Acquired immunity, which is supplied by lymphocyte functions, is the adaptive immune response and is crucial for in vivo infection prevention and external pathogen removal.^[13,18–20]^

Endogenous (liver: ~80–100 g and skeletal muscle: 300–400 g) glycogen stores are crucial because both moderate to high intensity (e.g., 65%–80% VO_2_ max) endurance activities and resistance-based workouts (e.g., 3–4 sets using ~6–20 repetition maximum loads) heavily rely on carbohydrates as a fuel source.^[21]^

Training for endurance combinations of carbohydrates and proteins are a classic tactic used by strength-power and endurance athletes to maximize exercise output, encourage glycogen repletion, reduce muscle breakdown, and maintain a positive nitrogen balance. Very few research have explicitly evaluated the effects of changing the time of when nutrients were provided. A small number of studies have looked at the effects of preendurance exercise ingestion of carbohydrate + protein on performance as well as metabolic consequences. There is evidence to suggest that a person’s health, ability to lose weight, or changes in body composition may be impacted by the time of day they consume the majority of their calories. First and foremost, it is crucial to emphasize that, with the exception of 2 recent papers involving trained men and women, the majority of the study on this topic has used nonathletic, untrained people.^[22,23]^ It remains to be seen if these results hold true for populations of highly skilled athletes. Study participants were mandated by Keim and colleagues to undergo two 6-week diet regimes that provided comparable macronutrient composition and calories (~1950 kcal). In a certain scenario, the participants had to eat 70% of their recommended daily consumption with breakfast, while in a different study group, they had to eat 70% of their recommended daily intake with dinner.^[24]^

The purpose of the Nutrition Examination Surveys is to track the nutritional status and general health of the civilian, noninstitutionalized population in the United States. The Centers for Disease Control and Prevention’s National Center for Health Statistics conducts the surveys.^[25]^ A test of submaximal treadmill exercise was included of the protocol. A recognized standard indicator of cardiorespiratory fitness is maximal oxygen consumption, or VO_2_ max, which is determined by gas exchange during strenuous activity. The submaximal treadmill test utilized in NHANES is a well-validated approach that uses the slope of the heart rate response to submaximal exercise to estimate VO_2_ max.^[26]^ Because of this, the information in this report offers a reliable assessment of the cardiorespiratory fitness levels of young people in modern America. These data are significant because it establish a foundation for tracking future developments in physical fitness among kids in the United States between the ages of 12 and 19. Furthermore, these data offer demographic norms that can be used to compare young people individually and in groups.

Numerous sports and activities include explosive movements and hard eccentric muscular contractions, which can cause varied degrees of muscle injury based on the individual’s training state, the intensity and length of the activity, and other factors. A summary of the mounting data showing that supplementing with fruit-derived polyphenols improves recovery from intense exercise, whether or not muscle injury occurs, and may even improve exercise performance was given by some studies.^[27]^

## 5. Conclusion

With strong publishing rates and centrality, the USA, People’s R China, and France have emerged as the 3 major research nations in this area. The strongest collaborations between developed nations and renowned institutions are beneficial to the advancement of sport and nutrition research. These articles were widely cited because they are a guideline or have a high impact factor.

## Acknowledgments

The authors would like to express their appreciation to Professor Chaomei Chen for inventing Citespace and making it free to use.

## Author contributions

**Conceptualization:** Wenqiang Wu.

**Data curation:** Wenqiang Wu.

**Formal analysis:** Wenqiang Wu.

**Funding acquisition:** Wenqiang Wu.

**Investigation:** Ye Tao.

**Methodology:** Ye Tao.

**Project administration:** Ye Tao.

**Resources:** Ye Tao.

**Software:** Ye Tao.

**Visualization:** Wenqiang Wu.

**Writing – original draft:** Wenqiang Wu.

**Writing – review & editing:** Wenqiang Wu.
